# Body satisfaction of female staff members working in primary schools in Mangaung, Bloemfontein

**DOI:** 10.4102/hsag.v26i0.1555

**Published:** 2021-03-30

**Authors:** Ntsoaki L. Meko, Mariette Nel

**Affiliations:** 1 Department of Nutrition and Dietetics, Faculty of Health Sciences, University of the Free State, Bloemfontein, South Africa; 2 Department of Biostatistics, Faculty of Health Sciences, University of the Free State, Bloemfontein, South Africa

**Keywords:** women, weight satisfaction, body mass index, weight management, body image

## Abstract

**Background:**

The role of psychosocial determinants of overweight and obesity is receiving attention in South African literature. South Africans tend to exhibit an inaccurate perception of their body weight.

**Aims:**

The purpose of this study was to determine levels of body satisfaction in female staff members working in primary schools in Mangaung, Bloemfontein.

**Setting:**

Mangaung is a peri-urban area in Bloemfontein in the Free State province of South Africa.

**Methods:**

Female staff members over the age of 18 years were invited to participate in the study. Anthropometric measurements of weight and height were measured using standardised techniques to calculate body mass index (BMI). Waist circumference (WC) was measured as an indicator of risk for non-communicable diseases (NCDs). Weight satisfaction was measured using a structured, self-administered body satisfaction questionnaire.

**Results:**

The majority of the staff members (71.3%) were classified as obese (BMI > 30 kg/m^2^); similarly a majority of them were at a high risk of NCDs according to WC. A little over a third (34.8%) of the women perceived themselves to have a normal weight. The majority of the women who had no concern with their body image were obese (59.1%) and only 8.7% of the women in this study were markedly concerned with their body image. Of the 60.9% of women who reported having attempted to lose weight, 38.6% reported using exercise and 30.0% used water as a weight loss method.

**Conclusion:**

Women’s awareness of a healthy weight should be promoted if efforts to achieve weight loss are to be effective.

## Introduction

The World Health Organization estimates that globally 1.9 billion people are overweight and of these 600 million are obese (WHO 2018). Adult overweight and obesity continue to rise as a result approximately half of the world’s population will be obese by 2030, even though obesity is preventable (Dobbs et al. 2014). South Africa (SA) has the highest prevalence of obesity in sub-Saharan Africa, with 68% of women being overweight or obese (NDOH et al. 2018). This increased burden of overweight and obesity in South African women is the cause for concern as it is a driver for the development for non-communicable diseases (NCDs) (Ford, Patel & Venkat Narayan 2017), which, in turn, translates to an increased disease burden (Reddy 2020).

Dietary factors and physical activity levels are the most common contributors to the development of overweight and obesity (Ford et al. 2017; Romieu et al. 2017). The determination of the role of psychosocial factors in the development of overweight and obesity, such as the presence of mental illnesses (e.g. anxiety and depression) and perceptions of body weight, has gained momentum (Halliwell 2015).

Burrows (2013) defined body satisfaction as the extent to which an individual is satisfied with his or her body shape, size and weight, whilst according to Tylka and Wood-Barcalow (2015) body image is a construct, which includes various complex and interrelated factors of how people experience their own physical appearance. Tylka and Wood-Barcalow (2015) defined a positive body image as the love, respect, acceptance and appreciation that a person has for his or her body. A positive body image is positively linked with intuitive eating and improved self-esteem (Tiggeman & McCourt 2013). A negative body image, on the other hand, is associated with depression, eating disorders and low self-esteem (Nikniaz et al. 2016).

Research on body image in SA has shown that women in the country do not perceive themselves as being too fat, nor are they concerned with their weight status (Prinsloo et al. 2011; Puoane et al. 2012). It seems from these studies that the overweight ideal, which is reported to reflect overweight and obesity as a sign of wealth, prosperity and well-being, persists (Puoane et al. 2012).

Research on body image in South African adults remains rare. Studies conducted in this field have mainly been conducted in the Western Cape Province (Puoane et al. 2012). A secondary analysis of the South African National Health and Nutrition Examination Survey (SANHANES-1) focused on body image perception in relation to weight status and weight management practices (Mchiza et al. 2015). Results from this and other surveys indicated here report that South Africans have an inaccurate perception of their body size (Mchiza et al. 2015; Okop, Levitt & Puoane 2019; Puoane et al. 2012).

On the contrary, studies on body image have focused mainly on adolescents in South Africa, comparing body dissatisfaction in boys and girls, as well as exploring differences in the prevalence of body dissatisfaction in girls across races and also based on whether they come from a rural or urban background (Gitau et al. 2014; Mchiza, Goedecke & Lambert 2011; Pedro et al. 2016). These studies reveal that black adolescent girls, particularly those from urban areas, show a tendency towards body dissatisfaction, a factor which was previously undocumented. In both black and white girls, body dissatisfaction takes the form of a ‘desire to be smaller’. This desire to be thin, however, is more dominant in white adolescent girls than in black girls (Gitau et al. 2014).

The continued burden of overweight and obesity in South Africa makes it critical that relevant intervention and prevention programmes are planned. Considering the diversity of the country, it is imperative that the extent of all factors leading to overweight and obesity is understood. The purpose of this article is to describe levels of weight satisfaction of women residing in Mangaung, Bloemfontein.

## Research methods and design

### Study design

A cross-sectional descriptive study was conducted as part of a larger study, which aimed to determine factors leading to overweight and obesity in women working in Mangaung Primary Schools.

### Study population and sampling

Permission to conduct the study was obtained from the Free State Department of Basic Education, as well as from the school principals of the selected schools. Female staff members in the selected schools were approached during a staff meeting and informed of the study. Female staff members working in primary schools in Mangaung, a peri-urban area in Bloemfontein in the Free State province, made up the study’s population. The names of the 30 primary schools in the area were entered in alphabetic order in a randomisation tool (Random.org: online), and 10 schools were randomly selected. The researcher made an appointment with the school principals to obtain permission for the school to be included in the study. Once approval was received, the aim of the study was explained to all female staff members working at the different schools. All female staff members over the age of 18 years, who were willing to participate in the study and who gave informed consent, were included in the study. Pregnant and lactating women were excluded from the study.

### Data collection methods

One classroom was assigned as a data collection room at each school. The researcher worked with two trained field workers to assist the participants in completing the self-administered questionnaires.

### Questionnaires

Socio-demographic information obtained from the participants in their preferred language (i.e. English or Southern Sesotho) included information on age, home language, marital status and education level. The women’s weight satisfaction was measured by determining their overall concern with their body shape using a body satisfaction questionnaire (Evans & Dolan 1993) divided into two sections. The first section of the questionnaire determined the women’s perception of their body weight and body weight control strategies. The second section consisted of eight questions, which determined how the women felt about their appearance (Evans & Dolan 1993). Each of the eight questions consisted of six possible answers. The scores to the answers were summed up and interpreted as having no concern, mild concern, moderate concern or marked concern with body image if the scores added up to < 19; 19–25; 26–33 and > 33, respectively.

### Anthropometry

All anthropometry was measured by the researcher using standardised techniques. Weight and height measurements were measured to calculate the women’s body mass index (BMI). Weight and height were measured with the women wearing minimal clothing (no heavy jackets) and no shoes. The measuring instruments were placed on a flat, hard level surface (Lee & Nieman 2013:170). Weight was measured using an electronic Tanita HD-327 scale with the women standing in the centre of the scale with feet evenly distributed on the platform. Height was measured using a Seca 214 portable stadiometer. The women were required to stand against the stadiometer with their heels, buttocks, shoulders and head touching its vertical surface. The women remained in a fully erect position, whilst the movable board was moved to the top of their head at the end of an expiration to take the measurement (Lee & Nieman 2013:170). The women were classified as underweight, normal weight, overweight or obese if their BMI was < 18.5 kg/m^2^, between 18.5 kg/m^2^ – 24.9 kg/m^2^, 25 kg/m^2^ – 29.9 kg/m^2^ and ≥ 30 kg/m^2^, respectively (WHO: online).

Waist circumference (WC), which is the narrowest circumference on the waist above the iliac crest and below the lower rib, was measured using a non-stretch measuring tape and used as an indicator of risk for NCDs (Katzmarzyk et al. 2011; Lee & Nieman 2013:183). Risk of NCDs was interpreted as increased if WC was between 80 cm and 87.9 cm and high if ≥ 88 cm.

### Statistical analysis

The Statistical Package for the Social Sciences (SPSS version 23) was used to analyse data. Descriptive statistics, namely, medians and percentiles, were calculated for continuous data, whilst frequencies and percentages used to present categorical data were calculated per group. The Fischer exact test was used to calculate the association between BMI and weight satisfaction. A *p*-value of < 0.05 was considered statistically significant.

### Ethical considerations

Approval for the study was obtained from the Health Sciences Research and Ethics Committee of the Faculty of Health Sciences at the University of the Free State (Ethical approval number [ETOVS NR.]71/2014). Collected data were kept confidential by allocating a unique number for each participant. Participation was voluntary and the participants could withdraw from the study at any time.

## Results

A total of 115 women working as educators (89.6%) and admin personnel (10.4%) in Mangaung schools participated in the study. The socio-demographic and anthropometric profile of the staff members who participated in the study are indicated in Tables 1 and 2, respectively. The women aged between 23 and 60 years were mostly Sesotho speaking (53.9%) and half of them (50.4%) were married. An unsurprising percentage (71.3%) of the women was classified as having a BMI of over 30 kg/m^2^, whilst only 10 women had a normal weight. A majority of the women (82.2%) had a high risk for NCDs as determined by a WC of over 88 cm.

**TABLE 1:** Socio-demographic status of female staff members.

Group	*n*	%
**First language**		
Sesotho	62	53.9
Setswana	45	39.1
isiXhosa	5	4.4
isiZulu	2	1.7
Other	1	0.9
**Marital**		
Single	29	25.2
Married	58	50.4
Divorced	16	13.9
Widowed	11	9.6
Other	1	0.9
**Job title**		
Educator	103	89.6
Admin clerk	12	10.4
**Highest level of education**		
Certificate	12	10.5
Diploma	40	35.1
Degree	62	54.4

**TABLE 2:** Body weight and waist circumference of female staff members.

Categories	*n*	%
**BMI categories (kg/m^2^)**		
Underweight (BMI < 18.5 kg/m^2^)	0	0
Normal weight (BMI 18 kg/m^2^ – 25 kg/m^2^)	10	8.7
Overweight (BMI 25 kg/m^2^ – 30 kg/m^2^)	23	20.0
Obese (BMI > 30 kg/m^2^)	82	71.3
**Waist circumference**		
No risk for NCDs (80 cm)	14	12.2
Increased risk for NCDs (80 cm – 88 cm)	18	15.6
High risk for NCDs (> than 88 cm)	83	72.2

BMI, body mass index; NCDs, non-communicable diseases.

**FIGURE 1:**
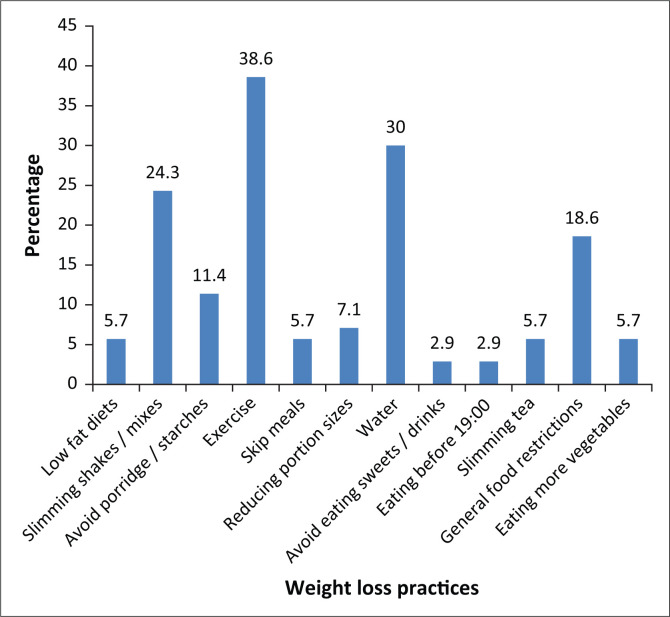
Weight loss practices of female staff members.

When asked about how they perceived their weight, only 38.3% of the women indicated being unhappy with their weight (Table 3). The women tended to underestimate their body sizes with a third (34.8%) considering themselves to be normal weight. Approximately 60.9% of the women indicated having tried to lose weight and the most common methods of weight loss used were exercising (38.6%), drinking water (30.0%) and using slimming shakes or mixes (24.3%) (Figure 1).

**TABLE 3:** Body perception of female staff members *n* (%).

Body perception questions	Happy		Somewhat happy		Unhappy		Underweight		Normal weight		Overweight		Yes		No	
	*n*	%	*n*	%	*n*	%	*n*	%	*n*	%	*n*	%	*n*	%	*n*	%
1. How happy are you with your present weight	27	23.5	44	38.3	44	38.3	-	-	-	-	-	-	-	-	-	-
2. Do you think you are	-	-	-	-	-	-	6	5.2	40	34.8	69	60	-	-	-	-
3. Have your family friends ever told you that you are fat	-	-	-	-	-	-	-	-	-	-	-	-	78	67.8	37	32.2
4. Have you ever tried to lose or trying to lose weight	-	-	-	-	-	-	-	-	-	-	-	-	70	60.9	45	39.1

When responses to the satisfaction questions were scored and categorised as indicated in Table 4, only 17.3% and 8.7% of the women had moderate and marked concern about their body image, respectively. The rest of the women had mild (35.7%) to no concern (38.3%) with their body image. Of the 38.3% of women who had no concern with their body image, the majority were obese (59.1%), whilst a quarter were overweight (25%). When the Fischer’s exact test was applied, BMI did not have an effect on body image (*p* = 0.1606).

**TABLE 4:** Body image satisfaction by body mass index of female staff members.

Satisfaction score	Total		Body weight (BMI, kg/m^2^)					
	*n*	%	Normal weight		Overweight		Obese	
			*n*	%	*n*	%	*n*	%
No concern	44	38.3	7	15.9	11	25.0	26	59.1
Mild concern	41	35.7	1	2.4	6	14.6	34	82.9
Moderate concern	20	17.3	2	10.0	5	25.0	13	65.0
Marked concern	10	8.7	0	-	1	10.0	9	90.0

BMI, body mass index.

Each of the eight questions included in the body satisfaction questionnaire was scored as never, rarely, sometimes, often, very often and always (Table 5). For ease of interpretation, the women’s responses were grouped as no concern if the response given was never and rarely, somewhat concerned for sometimes and often and concerned if the response was very often and always. Although the women were mostly somewhat concerned (46.9%) about their body shapes (60.8%), half of them did not feel self-conscious in situations such as taking a bath (54.8%) and most of them were not concerned about taking up too much room when they are around other people (73.1%). The women were also not concerned about other people seeing their rolls of fat around their waist or stomach (43.5%), nor did they avoid public places such as changing rooms or swimming pools where people could see their bodies (63.5%). Concern about their body image did, however, make 37.4% of the women and 35.7% somewhat and often feel that they should exercise, respectively.

**TABLE 5:** Body image satisfaction of female staff members.

Body image questions	Never		Rarely		Sometimes		Often		Very often		Always	
	No concern				Somewhat concerned				Concerned			
	*n*	%	*n*	%	*n*	%	*n*	%	*n*	%	*n*	%
Have you been worried about your shape?	34	29.6	11	9.6	52	45.2	2	1.7	3	2.6	13	11.3
Have you noticed the shape of other women and felt that yours doesn’t compare favourably?	39	33.9	11	9.6	47	40.9	5	4.4	7	6.1	6	5.2
Has being naked, such as when taking a bath made you feel self-conscious?	50	43.5	13	11.3	28	24.4	8	6.8	5	4.4	1 1	9.6
Have you worried about other people seeing rolls of fat around your waist or stomach?	40	34.8	10	8.7	32	27.8	8	6.9	9	7.8	16	13.9
Have you been worried about taking up too much room when you are around other people (e.g. sitting in a taxi, car, sofa, etc.)?	74	64.4	10	8.7	17	14.8	3	2.6	3	2.6	8	6.9
Have you avoided situations where people could see your body (e.g. communal changing rooms, swimming pools, etc.)?	53	46.1	20	17.4	26	22.6	4	3.5	0	0	12	10.4
Has eating sweets, cakes, or other high energy foods made you feel fat?	38	33.0	14	12.2	25	21.7	15	13.0	3	2.6	20	17.4
Has worry about your shape made you feel you should exercise?	24	20.9	7	6.1	27	23.5	16	13.9	13	11.3	28	24.4

## Discussion

This study mostly included educators who are seen as role models in the community. Other members of the female staff who participated in the study were school clerks. Most of the women were married and all of them had a post-matric qualification, meaning that this sample of women was well educated.

Overweight and obese individuals tend to underestimate their weight (Del Mar Bibiloni et al. 2017; Haynes et al. 2017; Mchiza et al. 2015) and this can partly be ascribed to the lack of understanding of what a healthy body weight is (Shisana et al. 2013). Only 8.7% of the women in this study had a normal BMI, whilst over 70% had a BMI indicative of obesity. Similar to other South African studies (Gradidge et al. 2015; Mchiza et al. 2015; Shisana et al. 2013) many of the women in this study underestimated their weight with just over a third of the women (35%) considering themselves to have a normal weight. The problem of body weight underestimation is not unique to South Africa. It is a common mismatch, which is found in both low-income (Bhanji et al. 2011) and middle- to high-income countries around the world (Robinson & Oldham 2016).

Even though the majority of the women in this study were overweight or obese (91.3%), most showed a mild to no concern with their body weight. Despite the fact that most of the women were happy about their body weight, they indicated being worried with their body shape (60.8%). The women’s body weight satisfaction, contrasted by a dissatisfaction with body shapes, is an indication that they may be dissatisfied with their appearance rather than their actual body weight (Tylka & Wood-Barcalow 2015). Whereas body weight dissatisfaction is reported to be high in overweight and obese individuals (Burrows 2013), this was not true for our study. Meanings associated with being overweight and obese in black populations are described in the literature (Puoane et al. 2012). In Western and white communities, an ‘ideal’ body shape is associated with social acceptance (Burrows 2013). This is different in African society where overweight is seen as culturally desirable, denoting beauty, affluence and prosperity and absence of the acquired immunodeficiency syndrome (AIDS) virus (Kruger 2018; Puoane et al. 2012). In the younger generation of black women, the trend may be changing to one where black girls now aim for slimmer body sizes, as influenced by the Western view of beauty (Gitau et al. 2014).

Pope, Corona and Belgrave (2014) suggested body appreciation and acceptance as possible reasons for weight satisfaction in overweight and obese women. A closer analysis of the women’s responses to questions in the body satisfaction questionnaire confirms this assumption. The women’s responses to the questions revealed 67.8% of the women having been told by family members that they were overweight. However, this did not seem to affect the women’s body image negatively as half of them (56.6%) did not consider themselves as inferior compared with other women, nor were they self-conscious in situations such as taking a bath, being in social spaces such as swimming pools or communal changing rooms. Over 70% were not concerned about taking up too much space when sitting around other people.

Body image dissatisfaction is associated with disordered eating, low self-esteem and depression (Tylka & Wood-Barcalow 2015). Although there is a slight increase in the prevalence of eating disorders in black populations, this is still low compared with white South African populations. Disordered eating was not apparent in this study; however, it is possible that worrying about their body shape prompted the study participants to engage in weight loss strategies. Approximately two-thirds (60.9%) of the women in this study reported that they had tried or that they were trying to lose weight. This high level of intention to lose weight is also a possible sign of women being more health conscious (Assari & Lankarani 2015), although this was not determined in this study. This percentage of women engaging in weight loss strategies is higher than what was found in the SANHANES-1 study where only 12% of South Africans attempted to lose weight (Mchiza et al. 2016) and it is also higher than in a study conducted in Beijing where 25% of the women reported taking action to lose weight (Cai et al. 2011).

Short-term attempts to lose weight are easy to achieve; however, weight loss maintenance presents a challenge (Sweeting & Caterson 2017:735). The most common weight management practices reported in our study, that is, engaging in physical activity, drinking more water, food restrictions, are similar to those reported in the secondary analysis of the SANHANES-1 study (Mchiza et al. 2015) and the Beijing study (Cai et al. 2011). Mangaung, like many other peri-urban areas in SA, has seen an increase in the number of community fitness clubs and gyms. It is therefore not surprising that most of the women trying to lose weight (38.6%) in our study reported having used exercise to do so. The use of exercise in weight loss has been proven to lead to good weight outcomes. Overall, only 27 women in this study reported using exercise as a strategy. Low levels of physical activity may be attributed to time-inconsistent features of behavior (Fan & Jin 2014). The authors propose that in the context of weight management, a present-biased preference determines how the benefits and costs of weight-related behaviours are evaluated. Actions such as exercise require what they refer to as immediate costs which result in delayed rewards; this, in turn, leads to individuals procrastinating and opting for actions which lead to immediate rewards instead (Fan & Jin 2014).

The second most common method of weight loss in this study was drinking water (30%). There is a prevailing belief in most townships that boiled water and boiled water with added lemon aids in weight loss. Using slimming shakes and slimming teas (24.4%), general food restrictions (18.6%) and avoiding maize porridge (11.7%) are other common methods used for weight loss. Societal pressure for thinness explains the use of commercial slimming shakes and slimming teas for weight loss. The use of shakes and teas is believed to facilitate more rapid weight loss, with the shakes offering approximately 1400 calories of energy per day (Cai et al. 2011). Avoidance of maize porridge is predominantly as a result of the belief that carbohydrate intake leads to the development of overweight and obesity.

Interpreting body satisfaction or lack thereof requires an understanding of body image, which does not just place positive and negative body image at opposing ends of one body image continuum. A more contemporary approach to body image is one which frames body image as a complex, multifaceted construct which extends beyond body satisfaction or appearance evaluation (Webb, Wood-Barcalow & Tylka 2015). Evaluation of body satisfaction needs to recognise this construct because it influences the way in which interventions are implemented.

It is important for health workers and programme planners to understand that people make decisions to improve their quality of life according to how they perceive their situation (Leonard et al. 2013). In the case of this study, lack of concern with body weight may mean that individuals take no action to correct their body weight, but would rather do so upon diagnosis with a life-threatening disease. Others may attempt to lose weight only to gain more weight with time (Haynes et al. 2017). Implementation of weight loss interventions in settings where women are not necessarily concerned about being overweight may require a mind-shift.

## Limitations of this study

As previously indicated, the field of body image and body satisfaction is one which is receiving increased attention. The most commonly used measure of body image perception in the literature are the Stunkard Figure Rating scales (Stunkard, Sorensen & Schulsinger 1983). This study, however, made use of a different measure developed by Evans and Dolan (1993) to determine body perception and body image satisfaction, meaning that comparisons with other studies are made with caution.

## Conclusion

Body weight underestimation was common in this study and the participants exhibited dissatisfaction with their body appearance. Interventions should focus their attention on education about a healthy body size in the form of a healthy BMI. In addition, communication strategies should address nutrition misinformation and should also aim to correct nutrition myths which lead to inappropriate weight loss strategies. Referral to dieticians, who are trained in providing evidence-based, individualised nutrition counselling should also be emphasised.
